# Synthesis of a Functionalized Polypyrrole Coated Electrotextile for Use in Biosensors

**DOI:** 10.3390/bios2040465

**Published:** 2012-11-29

**Authors:** Shannon K. McGraw, Evangelyn Alocilja, Andre Senecal, Kris Senecal

**Affiliations:** 1 Biosystems and Agricultural Engineering, Michigan State University, 524 S. Shaw Lane, 115 Farrall Hall, East Lansing, MI 48824, USA; E-Mail: mcgrawsh@msu.edu; 2 Food Protection Team, US Army Natick Soldier Research, Development, and Engineering Center (NSRDEC), Natick, MA 01760, USA; E-Mail: andre.g.senecal.civ@mail.mil; 3 Macromolecular Sciences and Engineering Team, US Army NSRDEC, Natick, MA 01760, USA; E-Mail: kris.j.senecal.civ@mail.mil

**Keywords:** electrotextile, biosensor, polypyrrole, immobilization

## Abstract

An electrotextile with a biosensing focus composed of conductive polymer coated microfibers that contain functional attachment sites for biorecognition elements was developed. Experiments were conducted to select a compound with a pendant functional group for inclusion in the polymer, a fiber platform, and polymerization solvent. The effects of dopant inclusion and post-polymerization wash steps were also analyzed. Finally, the successful attachment of avidin, which was then used to capture biotin, to the electrotextile was achieved. The initial results show a nonwoven fiber matrix can be successfully coated in a conductive, functionalized polymer while still maintaining surface area and fiber durability. A polypropylene fiber platform with a conductive polypyrrole coating using iron (III) chloride as an oxidant, water as a solvent, and 5-sulfosalicylic acid as a dopant exhibited the best coating consistency, material durability, and lowest resistance. Biological attachment of avidin was achieved on the fibers through the inclusion of a carboxyl functional group via 3-thiopheneacetic acid in the monomer. The immobilized avidin was then successfully used to capture biotin. This was confirmed through the use of fluorescent quantum dots and confocal microscopy. A preliminary electrochemical experiment using avidin for biotin detection was conducted. This technology will be extremely useful in the formation of electrotextiles for use in biosensor systems.

## 1. Introduction

Electrochemical biosensors combine a biological recognition element with an electrical readout. There is a large array of biorecognition elements to choose from including: DNA/RNA aptamers, proteins, antibodies, enzymes, and DNA probes. These biorecognition elements can be used directly in their natural form or can be biochemically altered [1,2,3,4]. Biological recognition is accomplished when the element is immobilized at an electrode transducer which converts the biological recognition event (*i.e*., antibody—antigen binding) into a measurable electrical signal [5,6,7,8]. Biosensor transducers can be electrochemical, optical, thermal, mass related, or based on electrical impedance. Impedance based sensing is advantageous because it does not require enzyme labels or reduction/oxidation mediators to facilitate detection [9]. In electrochemical impedance based biosensing, the biological recognition event creates a detectable disruption in the flow of either the electronic current or potential at the working electrode. 

High-surface area nonwoven fibers are versatile and can be developed into electrotextile smart membranes designed for use with all forms of sensor signal transduction. However, research into the integration of electrotextile, biological, and electrical technologies to create novel biosensor systems for food protection is limited. Previous studies have been conducted on the development of electrically active non-metallic textile coatings made of doped polypyrrole polymers [10,11,12,13,14,15]. It has been shown that an electrochemical biosensor electrode can be created by combining a conductive polymer coating on a non-woven microfiber support that is less expensive than its planar metal counterpart [16]. In addition, these electrotextile electrodes can be engineered to be durable, disposable, and require minimal attachment chemistries. With the attachment of biological recognition elements to the electrotextile surface, these electrodes have the capacity to act as the transducer in a biosensor while also performing pathogen capture, concentration, and detection [10]. This combination would simplify a food pathogen detection biosensor, resulting in a significantly smaller and lighter detection system.

The inclusion of a carboxyl group in the polymerization of such an electrotextile based sensor would provide the needed functional group sites for the attachment of biorecognition elements necessary to a biosensor design (*i.e*., antibodies, avidin). Various types of molecules have previously been included during the polymerization of pyrrole in order to create biosensors with built in biological receptor sites, such as biotin [17], benzophenone [18], pyrrole-3-carboxylic acid [19] and 3-thiopheneacetic acid [20]. Rapid, highly specific sensing of target analytes can be achieved due to the use of these elements at a relatively low cost. The faster speed and lower cost of biosensors *versus* standard detection methods have made them especially marketable to the food industry [21,22,23].

The objective of this study was to develop and produce an electrotextile with a biosensing focus composed of conductive polymer coated microfibers that contain functional attachment sites for biorecognition elements. Experiments were conducted to select a functional group, fiber platform, and polymerization solvent. The effects of dopant inclusion and post-polymerization wash steps were also analyzed. Finally, the successful attachment of avidin to the electrotextile was achieved, which was then used to capture biotin (a common biorecognition model). This was evaluated optically and electrochemically.

## 2. Experimental

### 2.1. Materials

Nylon 6 and polypropylene nonwoven microfibers were obtained from North Carolina State Nonwovens Cooperative Research Institute. The fibers were cut into circular discs with a diameter of 6.35 mm. The monomer solution contained 98% pyrrole and either 3-thiopheneacetic acid (3TAA) or pyrrole-3-carboxylic acid (3-COOH), all obtained from Sigma-Aldrich (St. Louis, MO, USA). Iron (III) chloride (FeCl_3_), acetonitrile, methanol, and 5-sulfosalicylic acid (5SSA) were also obtained from Sigma-Aldrich. Covalent attachment of the biorecognition elements was performed using N-(3-dimethylaminopropyl)-N*'*-ethylcarbodiimide hydrochloride (EDC) (Sigma-Aldrich) and N-hydroxysulfosuccinimide (sulfo-NHS) (Invitrogen, Carlsbad, CA, USA) with 50 mM 2-(N-morpholino)ethanesulfonic acid (MES) buffer, pH 6.0, (Thermo Fisher Scientific, Waltham, MA, USA), avidin (Thermo Fisher Scientific), biotin (Thermo Fisher Scientific), and biotinylated quantum dots (Qdot 655 biotin conjugate kit, Invitrogen). 

### 2.2. Synthesis

#### 2.2.1. Functional Group Selection

A circular nylon 6 membrane sample was dipped into a solution of 1 mg/mL 3TAA in pyrrole, removed, and placed into a reaction vessel where 10 mL 0.1 M FeCl_3_ in acetonitrile was added and allowed to react for 18 h at room temperature, during which oxidative polymerization of the pyrrole based monomer occurred [13,24,25,26,27]. The second sample was dipped into a solution of 0.5 mg/mL 3-COOH in pyrrole, removed, and placed into a container where 10 mL of 0.1 M FeCl_3_ in acetonitrile was added and allowed to react for 18 h at room temperature. Both samples were removed from their respective solutions and left to dry for 4 h at room temperature. 

#### 2.2.2. Dopant Inclusion and Solvent Selection

Six polymer samples were generated and evaluated using nylon 6 as the fiber platform. The fibers were dipped into a solution of 1 mg/mL 3TAA in pyrrole, removed, and placed into separate reaction vessels. Two samples each were oxidized with 10 mL of 0.1 M FeCl_3_ suspended in acetonitrile, methanol, or deionized (DI) water. A volume of 1 mL of 0.1 M 5SSA was added immediately after the addition of the FeCl3 to act as a dopant. All samples were allowed to react for 30 min at room temperature. After polymerization, the samples were removed and dried for 4 h at room temperature.

#### 2.2.3. Post-Polymerization Treatment

Nylon membrane samples were dipped into a solution of 1 mg/mL 3TAA, removed, and placed into an empty container. Iron chloride (10 mL, 0.1 M) was added to each followed immediately by 5SSA (1 mL, 0.1 M). Samples were allowed to react for 30 min. Samples were subject to 3 conditions: no post-polymerization treatment, a DI water wash, or a DI water wash and sonication for 5 min. Following treatment the samples were dried for 4 h at room temperature. 

#### 2.2.4. Fiber Platform Selection

A spot melted polypropylene disc was coated with the polypyrrole conductive polymer. The fiber disc was dipped into a solution of 1 mg/mL 3TAA in pyrrole, removed, and placed into a separate reaction vessel where polymerization occurred. A volume of 10 mL of 0.1 M FeCl_3_ in DI water was used as the oxidant and 1 mL of 0.1 M 5SSA was used as the dopant. The sample reacted for 30 min at room temperature and was then removed, washed with DI water, and dried for 4 h at room temperature. A sample using nylon 6 as the fiber platform was also prepared with an identical coating method.

### 2.3. Physicochemical Characterization

A visual assessment of each sample was conducted using scanning electron microscopy (SEM). The samples were gold sputter coated (~15 nm thickness) and imaged with a Zeiss EVO 60 scanning electron microscope (Carl Zeiss Microscopy, LLC, Thornwood, NY, USA). The images were taken at a setting of 1,024 × 768 pixels with 4× line integration (noise reduction technique). Slow scan speed 8 was used with a spot size of 370 for a measurement beam current of 70 picoamps. The EHT voltage was 30.0 kV and the working distance was 6 mm, except where noted otherwise. Electrical resistance measurements of the polymer were taken across the fiber membranes using a four point probe and a Keithley 2400 Sourcemeter (Keithley Instruments, Cleveland, OH, USA) after an interval of 10 s.

### 2.4. Biological Experiments

#### 2.4.1. Optical Analysis

Polypyrrole/3TAA coated polypropylene membrane discs were prepared according to the method listed in Section 2.2.4 and washed with DI water. Fluorescein isothiocyanate labeled avidin (FITC-avidin) was attached to the functionalized membranes through EDC/sulfo-NHS crosslinking. The discs were washed with distilled water and dried for 10 min. A volume of 200 µL of EDC and sulfo-NHS in MES buffer was added and reacted with gentle agitation for 15 min. The discs were washed twice in MES buffer and 250 µL of FITC-avidin was added to each disc. The discs were reacted with gentle agitation for 4 h and were washed with MES buffer. Finally, the discs were washed three times with phosphate buffered saline (PBS). A total volume of 500 µL biotinylated Qdots was added to the fibers at a 1:400 dilution. The samples were incubated with agitation for 1 h at room temperature and were stored at 4 °C overnight. The samples were washed three times with PBS and imaged using a Zeiss LSM710 confocal microscope (Carl Zeiss Microscopy, LLC, Thornwood, NY, USA).

#### 2.4.2. Electrochemical Analysis

Polypyrrole/3TAA coated polypropylene membrane discs were prepared according to the method listed in Section 2.2.4, cut into 2 cm × 2 cm squares, and washed with DI water. Avidin was attached to the functionalized membranes through EDC/sulfo-NHS crosslinking as described in Section 2.4.1. Resistance measurements were obtained by connecting the avidin attached membrane discs to a PalmSens using two alligator clips on opposing sides of the membrane. The membrane was submerged in 10 mL of 0.1 M phosphate buffer (PB) for 30 min in order to establish a baseline resistance for the fibers. After 30 min, a 10 mL sample of biotin solution (10 mL) of a particular concentration was added. The resistance values were recorded every 30 s for 15 min. The system response (Rp) was calculated using Equation (1) [10].

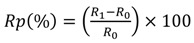
(1)
where *R*_1_ is the resistance of the avidin attached sensor after the biotin sample has been added. *R*_0_ is the resistance of the avidin attached sensor that has not been exposed to biotin.

## 3. Results and Discussion

Table 1 summarizes the different treatments that were examined in this manuscript as well as their impact on material resistance and coating morphology.

**Table 1:** Summary of treatments and effects.

Platform	Monomer	Dopant	Solvent	Reaction Time	Wash Treatment	Resistance	Figure #	Notes
Nylon 6	3-COOH/Pyrrole	none	acetonitrile	18 h	none	397 kΩ	1(A)	Coating even, black, and conformal on fibers. Heavy buildup of polymer clusters along fibers.
Nylon 6	3-TAA/Pyrrole	none	acetonitrile	18 h	none	23.71 kΩ	1(B)	Coating even, black, and conformal on fibers. Clusters of polymers buds scattered along fibers.
Nylon 6	3-TAA/Pyrrole	none	acetonitrile	30 min	none	189.98 kΩ	none	Coating uneven, gray, and brittle.
Nylon 6	3-TAA/Pyrrole	5SSA	acetonitrile	30 min	none	291 kΩ	none	Coating uneven, gray, with dark black spots, brittle.
Nylon 6	3-TAA/Pyrrole	none	methanol	30 min	none	35.31 MΩ	none	Coating uneven, gray, slight brittleness.
Nylon 6	3-TAA/Pyrrole	5SSA	methanol	30 min	none	710 Ω	2(A)	Coating uneven and gray with black spots. Polymer forms a solid sheet across fibers.
Nylon 6	3-TAA/Pyrrole	none	water	30 min	none	557 Ω	none	Coating smooth and even, black, slightly brittle
Nylon 6	3-TAA/Pyrrole	5SSA	water	30 min	none	91.5 Ω, 51.2 Ω	2(B)3(A)	Coating smooth, even, and black. Polymer clusters are small and build along fiber surfaces
Nylon 6	3-TAA/Pyrrole	5SSA	water	30 min	DI water wash and sonication	60.5 Ω	3(C)	Coating smooth, even, and black. Coating is slightly lighter than other samples with better porosity
Nylon 6	3-TAA/Pyrrole	5SSA	water	30 min	DI water wash	42.6 Ω	3(B)4(A)	Coating smooth, even, black, and slightly brittle. Coating is slightly lighter than unwashed sample
Polypropylene	3-TAA/Pyrrole	5SSA	water	30 min	DI water wash	55.1 Ω	4(B)	Coating smooth, even, and black. Not brittle. Coating is conformal along fibers.

### 3.1. Functional Group Selection

**Figure 1:**
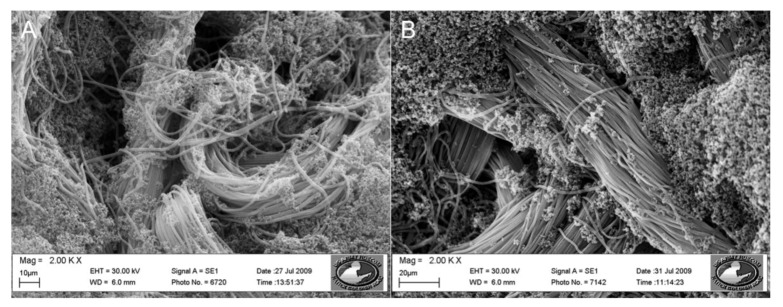
SEM images of fibers with 3-COOH *vs.* 3TAA in polymer coating. (**A**) Nylon 6 fibers coated in polypyrrole with 3-COOH. (**B**) Nylon 6 fibers coated in polypyrrole with 3TAA. Both at 2,000× magnification.

The inclusion of a pendant carboxyl functional group associated with the conductive polymer coating provides attachment sites for the covalent binding of antibodies to the fibers, giving a biosensor its ability to detect pathogens and specificity of capture [28]. The groups 3TAA and 3-COOH were evaluated as potential functional group additions in the electrotextile polymer. The SEM images of the samples can be seen in Figure 1 and the results can be seen in Table 1.

The sample using 3TAA formed an even black coating across the fiber surface. SEM analysis showed that the coating was conformal on the individual fibers on the membrane with clusters of polymer buds scattered along the fibers. The measured resistance for the sample was 23.71 kΩ. The sample with 3-COOH additive had an evenly dispersed black coating across the surface as well. SEM analysis showed that the fibers were conformally coated, however the buildup of polymer clusters on the fibers was much heavier than in the sample where 3TAA was used as the additive. This buildup of polymer caused an increased resistance of 397 kΩ for the sample. In the development of an electrotextile electrode, it is important to minimize material resistance and for the conductive polymer coating to be continuous throughout the fibrous platform. This ensures consistency across the electrode surface for recognition element attachment and that any change in electrical signal is due to target binding to the recognition site instead of variations between fabricated electrodes. Based on this information, 3TAA was selected as the functional group additive to be used in the polymerization.

**Scheme 1:**
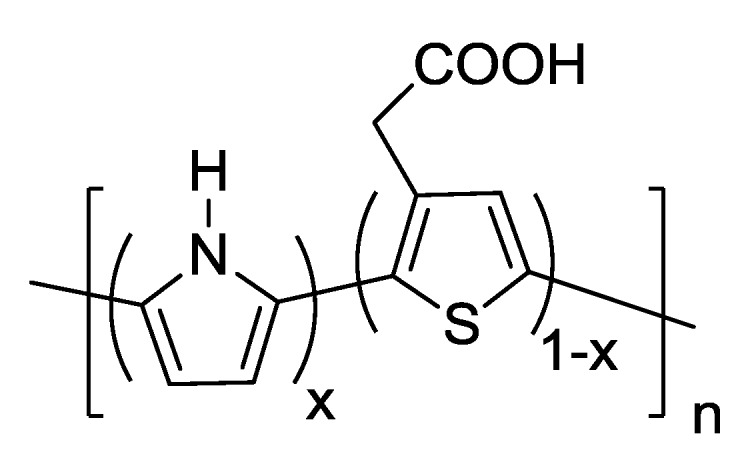
The structure of poly(pyrrole-3TAA).

The chemical structure of the poly(pyrrole-3TAA) copolymer can be seen in Scheme 1. Previous work has been done exploring the polymerization of pyrrole with additional molecules added to create a co-monomer in order to build biological receptor sites into the polymer. These include biotin [17], benzophenone [18], pyrrole-3-carboxylic acid (3-COOH) [19] and 3-thiopheneacetic acid (3TAA) [20]. The structure is the same as that published in Vaddiraju *et al.* [20], however because the deposition method is aqueous instead of oCVD there are differences in the coating thicknesses, morphologies, and conductivities. The addition of an organic acid dopant will also affect these parameters.

### 3.2. Dopant Inclusion and Solvent Selection

The inclusion of the dopant 5SSA was evaluated as a result of previous research indicating that the use of planar dopant ions increases conductivity in polypyrrole coatings [11,14,29]. The effect of the polymerization solvent was evaluated as well. These results can be seen in Table 1.

The samples with acetonitrile as a solvent were both unevenly coated, with the sample containing dopant having dark black spots across the surface. The coating was very brittle. As shown in Table 1, the measured resistance for the sample oxidized in acetonitrile with 5SSA was 291 kΩ. The resistance of the sample without the inclusion of the dopant was 189 kΩ. The samples where methanol was used as the solvent had uneven black coatings. The sample that did not have 5SSA added had a measured resistance of 35 MΩ, however the sample where 5SSA was added had a measured resistance of 710 Ω. 

The samples that were oxidized using FeCl_3_ in water had smooth and even black coatings. The sample without 5SSA had a slightly heavier surface coating and appeared more brittle. The sample without 5SSA had a measured resistance of 557 Ω. The sample with 5SSA had a resistance of 91.5 Ω.

**Figure 2:**
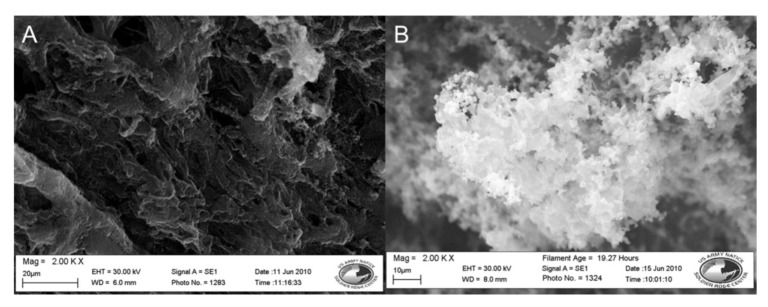
SEM images of nylon 6 fibers coated in doped polypyrrole, with methanol and water as solvents. (**A**) Methanol solvent, 2,000× magnification. (**B**) Water solvent, 2,000× magnification.

The samples containing 5SSA that were oxidized in methanol and in water were selected for further evaluation using an SEM. These images can be seen in Figure 2. The sample oxidized using methanol has a less globular appearance than previously seen in the acetonitrile samples without the dopant, however it appears more like a solid sheet of coating across the fibers. In comparison, the samples that were oxidized in water are very globular, the polymer clusters seen previously are present, but much smaller and building along each individual fiber.

When comparing the samples shown in Figure 2 with the samples from Figure 1, it can be seen that the choice of solvent for the reaction was shown to affect polymer formation on the fiber surface, directly relating to overall polymer conductivity. The inclusion of a dopant resulted in increased conductivity across the fiber membranes in less reaction time. The inclusion of the dopant seemed to have the greatest impact on conductivity when the conductive polymer was chemically oxidized in water resulting in the lowest sample resistance among the tested combinations.

### 3.3. Post-Polymerization Treatment

In order to evaluate the strength of the attachment of the coating to the fiber surface, a wash step and sonication were introduced post-polymerization to remove excess, unattached polymer. Each cleaning step was tested for effect on resistance. Untreated, washed, and sonicated samples were measured using a four point probe to determine the resistance. These results can be seen in Table 1. Their resistances were 51.2 Ω, 42.6 Ω, and 60.5 Ω, respectively. With a range of 17.9 Ω, no significant difference could be observed between the resistance measurements of the three samples. 

**Figure 3:**
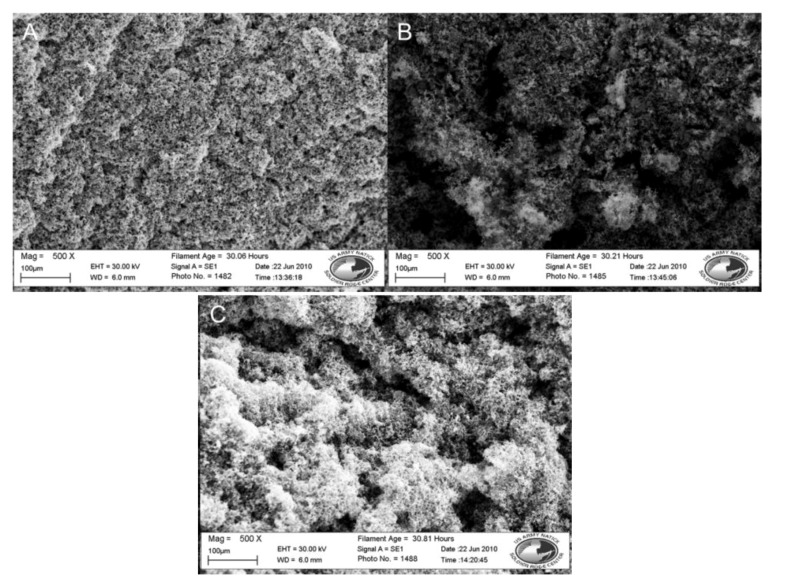
SEM images of nylon 6 fibers coated in doped polypyrrole with 3 post-polymerization treatments. (**A**) No rinse. (**B**) DI water wash. (**C**) DI water wash and sonication. All at 500×.

SEM analysis, shown in Figure 3, shows heavy, clustered polymer coatings along the fibers from each sample, with the sample that was not washed appearing to have a slightly heavier surface coating. The addition of a rinse step and sonication did not result in a significant loss of polymer coating from the fiber discs, however those samples did show better porosity between the individual fibers in the SEM images. The lack of a change in resistance and SEM images indicated that the polymer had bound to the nylon microfiber lattice. The larger clusters of polymer, where the polymer was attached to itself as opposed to the fiber surface, had weaker bonds, was removable and did not significantly change the resistance. Washing of the fibers post-polymerization was added to the protocol to allow for the removal of weakly bound excess, resulting in better fiber porosity and ensuring that the biorecognition elements would have access to the lower layers of the fiber mat.

### 3.4. Fiber Platform Selection

**Figure 4:**
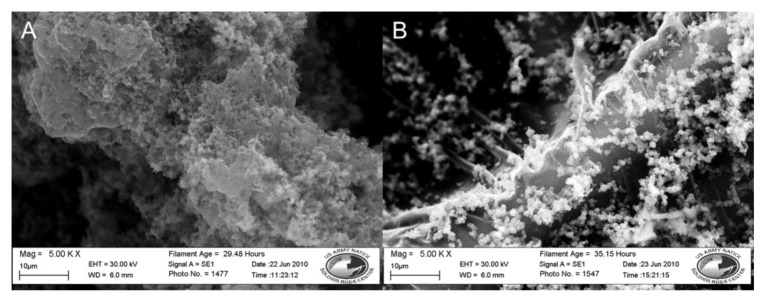
SEM images of nylon 6 *vs.* polypropylene fibers coated in doped polypyrrole. (**A**) Nylon 6. (**B**) Polypropylene. Both at 5,000×.

During drying the nylon coated fibers contracted, resulting in the disc becoming slightly smaller in diameter than before polymerization occurred. The coated fibers also became more brittle, occasionally fracturing when bent or twisted. To address this effect, a spot melted polypropylene disc, a more robust material, was coated with the polypyrrole conductive polymer using the procedure described in Section 2.2.4 in a 30 min reaction and compared to an identically coated nylon 6 membrane. After being washed with DI water SEM images of the samples were taken. These results can be seen in Table 1 and Figure 4.

The polypropylene disc in Figure 4 has an even black coating and a measured resistance of 55.1 Ω. The polypropylene microfibers are conformally coated with the polypyrrole polymer. The surface coating on the polypropylene fibers appears smoother than the coating on the nylon fibers. Nanoscale buds of polymer are seen scattered across the polymer surface ranging in size from 0.5 to 1 µm. At the areas where the nonwoven fibers are melted together, buds of polymer coating are seen in the range of 200 to 400 nm in diameter. The coated polypropylene discs also have better durability than its nylon counterpart. The polypropylene discs were able to be folded, rolled, and handled with less fracturing and loss of coating.

### 3.5. Biorecognition Element Attachment

#### 3.5.1. Optical Analysis

**Figure 5:**
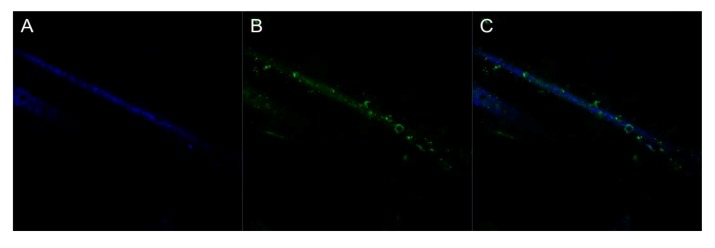
False colored confocal images of single fibers with FITC-avidin attachment. (**A**) Fiber reflectance. (**B**) Bound FITC-avidin. (**C**) Composite image. All at 4,000× with lasers at 405 and 488 nm.

**Figure 6:**
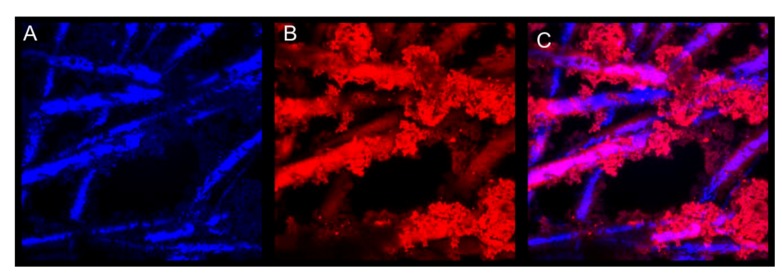
False colored confocal images of fibers with biotinylated quantum dot attachment. (**A**) Fiber reflectance. (**B**) Bound biotinylated quantum dots. (**C**) Composite image. All at 4,000× with lasers at 405 nm.

Generating a conductive polymer coating onto the fiber membranes has two purposes. The first is to make the fibers capable of conducting an electrical signal through the fibrous platform and the second is to provide attachment sites for biorecognition elements on these fibrous surfaces. Confocal microscopy was used to determine if FITC labeled avidin was covalently bound to the polymer coating. Qdot labeled biotin was then used to indicate if the surface bound avidin had maintained its capture ability. Figure 5 and Figure 6 confirm the presence of functional groups for bio-recognition attachment in the polymer. Figure 5 shows a coated fiber that has FITC-labeled avidin attached to it. Figure 6 shows the same fiber sample, after the addition of Qdot labeled biotin. The avidin has bound to the polymer coating and then conjugated with the biotin. This indicates that a biorecognition element, avidin, can be successfully bound to the polymer coating using covalent attachment chemistry and can be used to perform capture.

#### 3.5.2. Electrochemical Analysis

**Figure 7:**
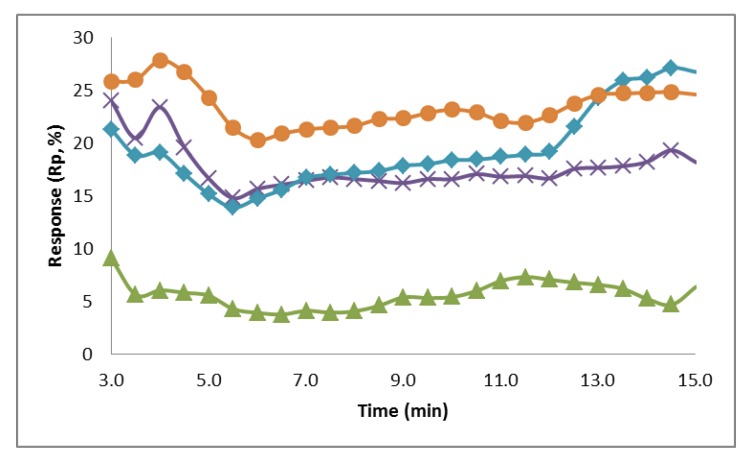
Average response over a period of 12 min (after a 3 min equilibrium time) for the electrochemical detection with varying biotin solution concentrations of 0.5 µM (green, triangle), 5 µM (purple, x), 50 µM (blue, diamond), and 500 µM (orange, circle). The system response increases as the concentration of biotin increases.

Preliminary experiments were conducted taking multiple measurements using the fiber membranes as electrodes to determine if a biological recognition signal can be observed. Triplicate measurements were taken using the conductive fiber membrane electrodes to establish the resistance values for a control sample (0.1 M PB) and biotin solutions at concentrations of 0.5, 5, 50, and 500 µM. A time of 3 min was determined to be necessary to reach system equilibrium. The responses for each concentration at each time point were averaged and the value after the initial 3 min equilibrium time can be seen in Figure 7.

The response of the system at each sample concentration was plotted over the 12 min period, showing that the system response increases as the concentration of biotin increases. The average percent response at a concentration of 0.5 µM was 5.6%. For the biotin concentrations of 5, 50, and 500 µM the average responses were 17.7%, 19.4%, and 23.4%, respectively. Figure 7 shows that the responses at concentrations of 5 µM, 50 µM, and 500 µM were all significantly larger than the response at 0.5 µM. Also, the responses at these three concentrations are very close, with the response at 5 and 50 µM crossing at the 7 min mark and between 50 and 500 µM at 13 min. This is most likely due to the fibers reaching a threshold for attachment on the surface, so that the increase of biotin in the sample is no longer generating a proportional increase on the system resistance. The surface attachment capabilities could be improved by increasing the size and therefore surface area of the fiber mats, attaching a higher concentration of avidin to the fibers, and increasing the amount of carboxyl group attachment sites on the fibers.

The resistance of the sensor at each biotin solution concentration was tested against the values of the control using a Student’s t-Test (1 tail, α = 0.05) to determine significance. The resistance at each concentration of biotin tested was determined to be significantly different from the blank. The Student’s t-Test was also used to determine that, after 3 min, the resistances measured at each concentration were significantly different than at every other tested concentration. This shows that the electrotextile electrode is capable of differentiating between small changes in conductivity due to the addition of biotin to the system. It also has the potential to eventually be used as a simple sample capture and read system for pathogen detection.

## 4. Conclusions

The long-term goal of this research is to develop a rapid and novel electrochemical biosensor for the detection of pathogens. Ultimately, the nonwoven membrane platform will be capable of being immersed into a test sample to act as a pathogen collector and then inserted into an electrochemical cell to complete the biosensor circuit by functioning as the working electrode.

The initial results from this study show a nonwoven fiber matrix can be successfully coated in a conductive, functionalized polymer while still maintaining surface area and fiber durability. A polypropylene fiber platform with a conductive polypyrrole coating using FeCl_3_ as an oxidant, water as a solvent, and 5SSA as a dopant exhibited the best coating consistency, material durability, and lowest resistance. Furthermore, biological attachment using avidin-biotin can be achieved on the fibers through the inclusion of a carboxyl functional group via 3TAA in the monomer. When put into a simple electrochemical system, the membranes could be used to successfully detect biotin in solution at concentrations of 0.5, 5, 50, and 500 µM. This technology will be extremely useful in the formation of electrotextiles for use in biosensor systems. 

Future research will explore the effects of each polymerization component, different fiber platforms, and the attachment of other biorecognition elements to the coated fiber surfaces.
